# Sex Differences in Poststroke Cognitive Impairment: A Multicenter Study in 2343 Patients With Acute Ischemic Stroke

**DOI:** 10.1161/STROKEAHA.123.042507

**Published:** 2023-08-08

**Authors:** Lieza G. Exalto, Nick A. Weaver, Hugo J. Kuijf, Hugo P. Aben, Hee-Joon Bae, Jonathan G. Best, Régis Bordet, Christopher P.L.H. Chen, Ruben S. van der Giessen, Olivier Godefroy, Bibek Gyanwali, Olivia K.L. Hamilton, Saima Hilal, Irene M.C. Huenges Wajer, Jonguk Kim, L. Jaap Kappelle, Beom Joon Kim, Sebastian Köhler, Paul L.M. de Kort, Peter J. Koudstaal, Jae-Sung Lim, Stephen D.J. Makin, Vincent C.T. Mok, Robert J. van Oostenbrugge, Martine Roussel, Julie Staals, Maria del C. Valdés-Hernández, Narayanaswamy Venketasubramanian, Frans R.J. Verhey, Joanna M. Wardlaw, David J. Werring, Xin Xu, Martine J.E. van Zandvoort, J. Matthijs Biesbroek, Francesca M. Chappell, Geert Jan Biessels

**Affiliations:** 1 Department of Neurology and Neurosurgery, UMC Utrecht Brain Center, the Netherlands (L.G.E., N.A.W., I.M.C.H.W., L.J.K., M.J.E.v.Z., J.M.B., G.J.B.).; 2 Image Sciences Institute, University Medical Center Utrecht, the Netherlands (H.J.K.).; 3 Department of Neurology, Elisabeth Tweesteden Hospital, Tilburg, the Netherlands (H.P.A., P.L.M.d.K.).; 4 Department of Neurology, Seoul National University Bundang Hospital, Seoul National University College of Medicine, Seongnam, Republic of Korea (H.-J.B., J.K., B.J.K.).; 5 Stroke Research Centre, Department of Brain Repair and Rehabilitation, UCL Queen Square Institute of Neurology, London, United Kingdom (J.G.B., D.J.W.).; 6 Université Lille, Inserm, CHU Lille, U1172 - LilNCog - Lille Neuroscience & Cognition, F-Lille, France (R.B.).; 7 Department of Pharmacology, National University of Singapore (C.P.L.H.C., S.H., X.X.).; 8 Memory, Aging and Cognition Center, National University Health System, Singapore, Singapore (C.P.L.H.C., B.G., S.H., X.X.).; 9 Department of Neurology, Erasmus Medical Center, Rotterdam, the Netherlands (R.S.v.d.G., P.J.K.).; 10 Department of Neurology, Amiens University hospital, Laboratory of Functional Neurosciences, Jules Verne Picardy University, France (O.G., M.R.).; 11 Neuroimaging Sciences, Centre for Clinical Brain Sciences, University of Edinburgh, United Kingdom (O.K.L.H., M.d.C.V.-H., J.M.W.).; 12 UK Dementia Research Institute at the University of Edinburgh, United Kingdom (O.K.L.H., M.d.C.V.-H., J.M.W.).; 13 Saw Swee Hock School of Public Health, National University of Singapore and National University Health System (S.H.).; 14 Experimental Psychology, Helmholtz Institute, Utrecht University, the Netherlands (I.M.C.H.W., M.J.E.v.Z.).; 15 Department of Psychiatry and Neuropsychology, School for Mental Health and Neuroscience, Maastricht University, the Netherlands (S.K., F.R.J.V.).; 16 Department of Neurology, Asan Medical Center, University of Ulsan College of Medicine, Seoul, Republic of Korea (J.-S.L.).; 17 Centre For Rural Health, Institute of Applied Health Sciences, University of Aberdeen, United Kingdom (S.D.J.M.).; 18 Division of Neurology, Department of Medicine and Therapeutics (V.C.T.M.), The Chinese University of Hong Kong.; 19 Therese Pei Fong Chow Research Centre for Prevention of Dementia, Margaret Kam Ling Cheung Research Centre for Management of Parkinsonism, Gerald Choa Neuroscience Centre (V.C.T.M.), The Chinese University of Hong Kong.; 20 Department of Neurology, Maastricht University Medical Center, the Netherlands (R.J.v.O., J.S.).; 21 Raffles Neuroscience Centre, Raffles Hospital, Singapore, Singapore (N.V.).; 22 Department of Neurology, Diakonessenhuis Hospital, Utrecht, the Netherlands (J.M.B.).; 23 Social and Public Health Sciences Unit (F.M.C.), University of Glasgow, United Kingdom.; 24 MRC/CSO Social and Public Health Sciences Unit, School of Health and Wellbeing (O.K.L.H.), University of Glasgow, United Kingdom.

**Keywords:** cognition, ischemic stroke, men, survivors, women

## Abstract

**BACKGROUND:**

Poststroke cognitive impairment (PSCI) occurs in about half of stroke survivors. Cumulative evidence indicates that functional outcomes of stroke are worse in women than men. Yet it is unknown whether the occurrence and characteristics of PSCI differ between men and women.

**METHODS:**

Individual patient data from 9 cohorts of patients with ischemic stroke were harmonized and pooled through the Meta-VCI-Map consortium (n=2343, 38% women). We included patients with visible symptomatic infarcts on computed tomography/magnetic resonance imaging and cognitive assessment within 15 months after stroke. PSCI was defined as impairment in ≥1 cognitive domains on neuropsychological assessment. Logistic regression analyses were performed to compare men to women, adjusted for study cohort, to obtain odds ratios for PSCI and individual cognitive domains. We also explored sensitivity and specificity of cognitive screening tools for detecting PSCI, according to sex (Mini-Mental State Examination, 4 cohorts, n=1814; Montreal Cognitive Assessment, 3 cohorts, n=278).

**RESULTS:**

PSCI was found in 51% of both women and men. Men had a lower risk of impairment of attention and executive functioning (men: odds ratio, 0.76 [95% CI, 0.61–0.96]), and language (men: odds ratio, 0.67 [95% CI, 0.45–0.85]), but a higher risk of verbal memory impairment (men: odds ratio, 1.43 [95% CI, 1.17–1.75]). The sensitivity of Mini-Mental State Examination (<25) for PSCI was higher for women (0.53) than for men (0.27; *P*=0.02), with a lower specificity for women (0.80) than men (0.96; *P*=0.01). Sensitivity and specificity of Montreal Cognitive Assessment (<26.) for PSCI was comparable between women and men (0.91 versus 0.86; *P*=0.62 and 0.29 versus 0.28; *P*=0.86, respectively).

**CONCLUSIONS:**

Sex was not associated with PSCI occurrence but affected domains differed between men and women. The latter may explain why sensitivity of the Mini-Mental State Examination for detecting PSCI was higher in women with a lower specificity compared with men. These sex differences need to be considered when screening for and diagnosing PSCI in clinical practice.

Poststroke cognitive impairment (PSCI) is a common consequence of ischemic stroke, and a leading cause of long-term disability and reduced quality of life.^1^ PSCI occurs in approximately half of stroke survivors within the first year.^2,3^ Early detection of PSCI can facilitate tailored rehabilitation and informed planning for long-term needs.^4^

Accumulative evidence indicates that functional outcomes of stroke are worse in women compared with men. A review on sex differences in stroke outcomes showed that women experience more activity limitations, worse quality of life, and more poststroke depression than men.^5^ Whether women and men differ with regard to PSCI is less clear,^5^ since only limited and heterogeneous studies are available,^6–10^ which differ in the definition of PSCI and cognitive assessment protocols. Most studies used a global measure of cognitive functioning, measured with screening tests like the Montreal Cognitive Assessment (MoCA). This reflects clinical practice, as a screening test is more readily available and quicker than multidomain cognitive assessment. Moreover, it remains unclear whether the cognitive profile of PSCI, that is, affected cognitive domains, differs between women and men. In Alzheimer dementia, differences in cognitive profile between women and men have previously been shown, therefore such differences might also apply to other forms of cognitive impairment, such as PSCI.^11^

We aimed to investigate sex differences in the occurrence and type of PSCI in patients with acute ischemic stroke. As a secondary objective, we explored sex differences in the sensitivity and specificity of cognitive screening tools to identify PSCI.

## METHODS

The data that support the findings of this study are available from the corresponding author on reasonable request.

### Study Design and Participants

**Figure 1.**
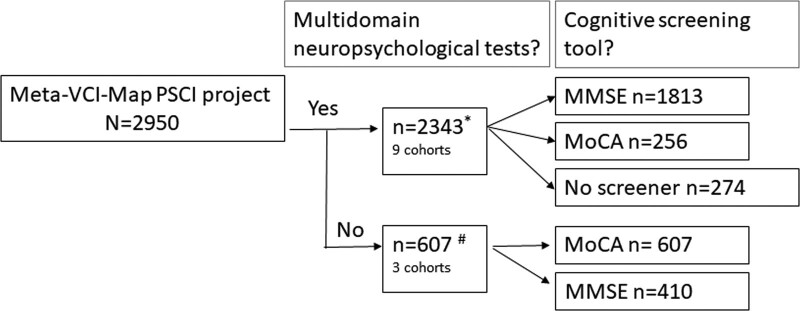
**Number of performed cognitive screening tools in relation to performed multidomain neuropsychological tests.** *Pooled data for main analysis. #Can have both Montreal Cognitive Assessment (MoCA) and Mini-Mental State Examination (MMSE). PSCI indicates poststroke cognitive impairment.

Participants were identified through the Meta-VCI-Map consortium (http://www.metavcimap.org/; for details see design paper^12^); an international collaborative platform for lesion-symptom mapping studies initiated in 2018 and still expanding. For the current project, 12 cohorts (as of January 1, 2029) were identified based on: (1) cohort of patients with acute ischemic stroke; (2) brain computed tomography or magnetic resonance imaging (T1/T2/FLAIR/DWI) showing the symptomatic infarct(s); (3) available infarct segmentations; and (4) cognitive assessment within 15 months after stroke. Figure 1 shows the number of performed cognitive screening tools in relation to performed multidomain neuropsychological tests of eligible patients for the current study. For the main analysis, only patients with a multidomain neuropsychological assessment were selected. The data of 9 cohorts (n=2343) from France (N=2), Hong Kong (N=1), the Republic of Korea (N=2), the Netherlands (N=3), and Singapore (N=1), were pooled. For supplementary analyses on cognitive screening instruments, 3 additional cohorts ([n=607] from the Netherlands [n=1], and the United Kingdom [N=2]) were identified with a cognitive screening test (Mini-Mental State Examination [MMSE] or MoCA), but without multidomain neuropsychological assessment. In all patients, language ability had to be sufficiently preserved to undergo cognitive testing. Inclusion and exclusion criteria of participating cohorts are presented in Table S1. Data processing and analysis were performed at the University Medical Center Utrecht. The article adheres to the observational cohort guideline.

### Participant Characteristics

Several sociodemographic and clinical characteristics were collected (Table 1). All participating cohorts provided level of education, however using different methods, for example, years or categories. Harmonization of educational level data was done as described previously,^13^ by recoding the original education data into a 4-category variable according to the approach in the Stroke and Cognition (STROKOG) consortium.^14^ Severity of stroke was measured by the National Institutes of Health Stroke Scale.^15^ Several cohorts used the Informant Questionnaire on Cognitive Decline in the Elderly to assess prestroke cognitive decline.^16^ For the current study, infarct type was divided into small subcortical infarcts versus other infarct type, which are mutually exclusive. Small subcortical infarcts were defined as single supratentorial infarcts without cortical involvement, with a lesion volume of ≤4.19 mL (ie, a sphere of ≤2 cm diameter; following the Standards for Reporting Vascular Changes on Neuroimaging criteria^17^). Lesion volume was based on infarct segmentation methods, as previously published.^13^

**Table 1.**
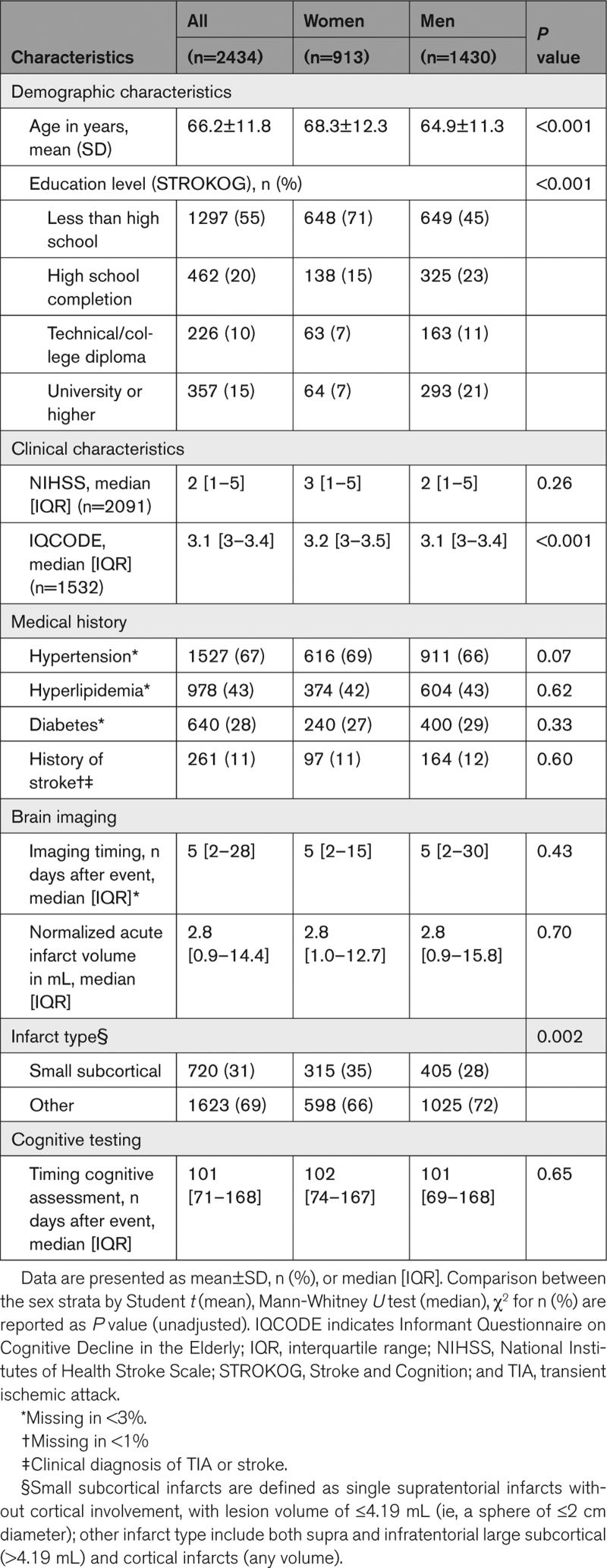
Baseline Characteristics Total Study Sample and Stratified by Sex

### Cognitive Data Harmonization

The neuropsychological test battery (NTB) to assess cognitive performance differed by cohort (Table S2) and was generally performed 2 to 6 months poststroke (mean 117 days, SD, 79). The harmonization of the cognitive data has been described in detail previously.^18^ Cognitive tests from each cohort were assigned to 6 cognitive domains: (1) attention and executive functioning; (2) information processing speed; (3) language; (4) verbal memory; (5) visuospatial perception/construction; and (6) visuospatial memory. Only tests with (local) norm-referenced data were used. Assignment to cognitive domains was based on previous work^19^ and reviewed by a neuropsychology working group.^13^ An overview of normative data per cohort is provided in the appendix (Table S3). For each test performance, <5th percentile was defined as impaired. Performance on a cognitive domain was impaired if >50% of available neuropsychological tests on that domain were impaired, which was determined on a per-subject basis (ie, patients might have a different number of available tests available per domain). PSCI was defined as cognitive impairment in ≥1 cognitive domains, in accordance with the VASCOG criteria for Vascular Cognitive Disorders.^20^ The number of available tests and domains could differ between cohorts. To be able to rule out PSCI at patient level, data on a minimum of 3 cognitive domains needed to be available. The percentage of patients assessed per domain ranged between 34% and 98% (Table 2). With regard to cognitive screening tools in the 9 cohorts with a multidomain neuropsychological assessment, MMSE was utilized in 4 cohorts (n=1814, missing score n=1), MoCA in 3 other cohorts (n=278, missing score n=22), and no cognitive screening test was available in the other 2 cohorts (n=251).

**Table 2.**
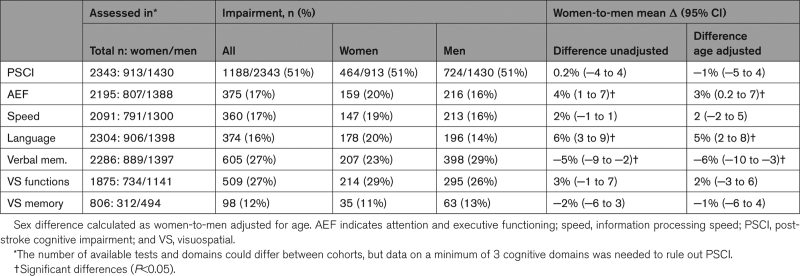
PSCI and Impairment of the Cognitive Domains

### Ethics Statement

Participating centers adhered to their local regulations for data deidentification and data sharing and obtained ethical approval from their local institutional review boards before participation.

### Statistics

Women and men were compared using independent samples *t* tests for parametric data, Mann-Whitney *U* test for nonparametric data, and χ^2^ tests for proportions. We used general linear models to obtain age-adjusted women-to-men differences and 95% CI for characteristics of cognitive impairment.

Logistic regression analyses with cohort as covariate were performed to obtain odds ratios (ORs) for PSCI and impairments in the separate cognitive domains; women denoted the reference group. To identify PSCI, norm-referenced data was used to express cognitive performance in percentiles. This normative data was already corrected for age, level of education and sex, therefore we did not add these covariates to the regression analyses. To investigate the potential influence of age, education, and infarct type in further detail, the analyses were repeated stratified by age (<65 versus ≥65 years), education (<high school versus ≥high school), and infarct type (small subcortical versus other infarct type). In addition, we evaluated a putative interaction with the 3 aforementioned stratification variables with sex by adding an interaction-term (eg, sex×age<65 years) to separate models. Sensitivity analyses on the risk of PSCI per cohort were performed (Figure S1). All of the above mentioned analyses were done in SPSS 26.

For the meta-analysis of the sensitivity and specificity of cognitive screening tools, only cohorts with both NTB and MoCA (n=3) or NTB and MMSE (n=4) were used. PSCI was diagnosed on the above-described criteria of NTB. The standard cutoffs were used for the MMSE^21^ (<25) and for MoCA^22^ (<26.) The Cochrane Handbook for Systematic Reviews of Diagnostic Test Accuracy was used for the analysis.^23^ Heterogeneity is to be expected in meta‐analyses of diagnostic test accuracy.^24^ First, forest plots were created and evaluated for heterogeneity. Next, a ROC plot was created to evaluate potential threshold effect. Where the ROC plot showed evidence of a threshold effect, the meta-analyses used the bivariate model, and where there was little evidence, separate analyses for sensitivity and specificity. In all cases, the within-study variance was modeled as binomial.^23^ SAS 9.4 (www.sas.com) was used for the analysis, and R 3.6.2 (https://cran.r-project.org/) for the graphs.

Statistical significance was based on threshold *P*<0.05.

This article follows the STROBE reporting guideline.^25^

## RESULTS

We included 2434 patients, 38% of whom were women, from 9 cohorts. Baseline characteristics for the total sample and stratified by sex are presented in Table 1 and per cohort in Table S4. Women were on average older (mean age, 68.3; SD, 12.3 years versus 64.9 SD, 11.3 years) and lower educated than men (71% <high school versus 45%). Stroke severity was higher in women than men (National Institutes of Health Stroke Scale: median, 3 [interquartile range, 1–5] versus 2 [interquartile range, 1–5]). The scores on the Informant Questionnaire on Cognitive Decline in the Elderly were also higher in women than men (median, 3.2 [interquartile range, 3–3.5] versus 3.1 [interquartile range, 3–3.4]). The acute lesion was more commonly a small subcortical infarct in women (35% compared with men 28%), whereas consequently, other types of infarcts (large subcortical, cortical, or infratentorial) were more common in men (72% compared with women 66%) There was no difference between men and women with respect to history of previous stroke (11 versus 12%), nor the median time to imaging, (5 days) or cognitive testing (101 days, Table S5).

**Figure 2.**
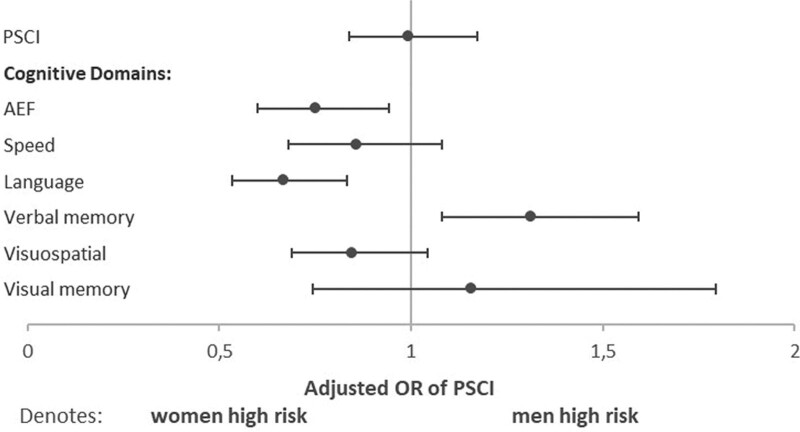
**Sex differences in risk of poststroke cognitive impairment (PSCI) and impaired cognitive domains.** Logistic regression analyses were performed to obtain odds ratio (OR) for PSCI with covariate study cohort. AEF indicates attention and executive functioning; speed, information processing speed; and visuospatial, visuospatial perception/construction.

**Figure 3.**
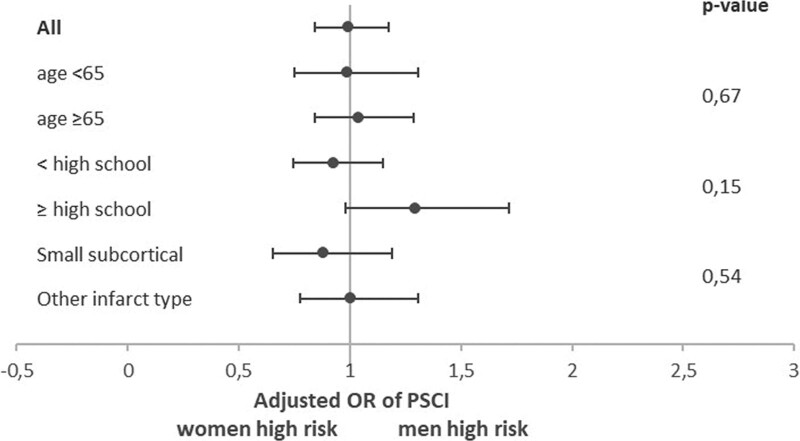
**Sex differences in risk of poststroke cognitive impairment (PSCI) stratified by demographics and infarct type.** Logistic regression analyses were performed to obtain odds ratio (OR) for PSCI with covariate study cohort. The *P* values correspond to the interaction term, so stratification-by-sex interaction, for example, age×sex.

PSCI was found in 51% of both women and men (age-adjusted women-to-men differences, −1% [95% CI, −5 to 4). Women and men were equally likely to have PSCI (OR, 1.03 [95% CI, 0.87–1.21]; Figure 2). Different cognitive domains were impaired in variable frequencies, ranging from 11% to 29% in women and 13% to 29% in men (Table 2). The most commonly affected domain was visuospatial perception/construction for women and verbal memory for men. Men had a lower risk of impairment in the cognitive domains of attention and executive functioning (OR, 0.76 [95% CI, 0.61–0.96]), and language (OR, 0.67 [95% CI, 0.54–0.85]) than women but a higher risk of impairment in verbal memory (OR, 1.43 (95% CI, 1.17–1.75; Figure 2). The risk of impairment in the other domains (information processing speed, language, visuospatial perception/construction, and visuospatial memory) was comparable between the sexes. There was no significant interaction between sex and age, level of education, or type of stroke on PSCI occurrence. Figure 3 shows the risk of PSCI between the sexes stratified by age, level of education, and type of infarct (similar analyses for the 6 cognitive domains are shown in Figure S2).

**Figure 4.**
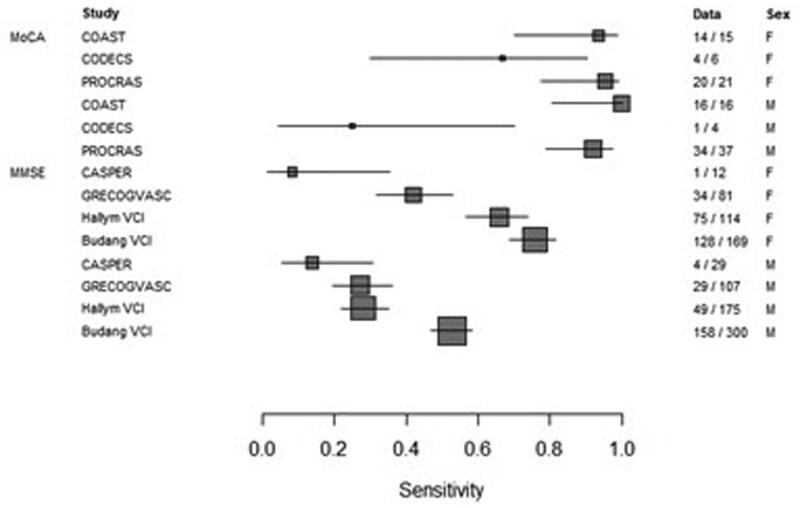
**Sensitivity of Montreal Cognitive Assessment (MoCA) and Mini-Mental State Examination (MMSE) by sex.** Forrest plots based on random effects model.

**Figure 5.**
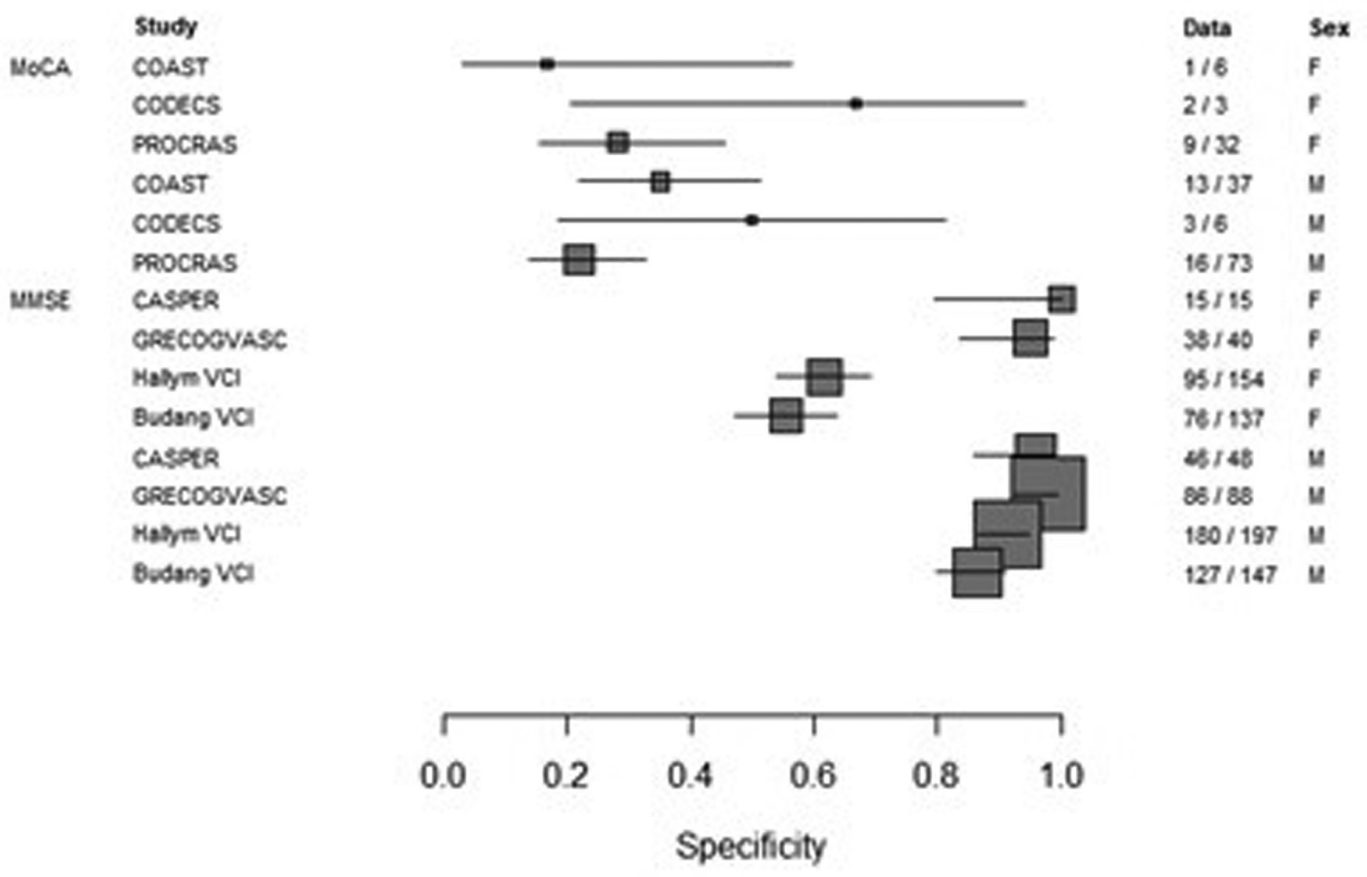
**Specificity of Montreal Cognitive Assessment (MoCA) and Mini-Mental State Examination (MMSE) by sex.** Forrest plots based on random effects model.

The meta-analysis of sensitivity and specificity of the cognitive screening tools, MMSE (n=1813) and MoCA (n=256), for PSCI by sex are presented in forest plots in Figures 4 and 5. The heterogeneity in sensitivity seen in the forest plots for the MMSE is due to a threshold effect (ROC curve in Figure S3). The sensitivity and specificity of MoCA for PSCI did not differ between women and men (0.91 versus 0.86; *P*=0.62 and 0.29 versus 0.28; *P*=0.86, respectively). The sensitivity of MMSE for PSCI is higher in women (0.53) compared with men (0.27; *P*=0.02). The specificity of MMSE for PSCI was lower in women (0.80) compared with men (0.96; *P*=0.01). Details on this analysis are shown in Table S6. The MMSE score was below the cutoff for impairment in 63% of women with PSCI and in 39% of men with PSCI. In the group without PSCI, 35% of the women and 9% of the men scored below the MMSE cutoff. The MoCA score was below the cutoff for impairment in 91% of women with PSCI and 90% of men with PSCI. In the group without PSCI, the MoCA score was below the cutoff in 71% of women and in 70% of men (Table S7).

In the 3 supplementary cohorts without NTB (n=607), but with a MMSE (n=410) or MoCA (n=607), women were (in line with the main cohort) older and less educated compared with men (Table S8). MoCA score was below the cutoff for impairment in 84% of women and 66% of men (*P*<0.000). The MMSE score was below the cutoff for impairment in 48% of women and 52% of men (*P*=0.39; Table S5).

## DISCUSSION

In this large multicenter study, we found that PSCI was equally common in women and men, but that the cognitive profiles differed between sexes. Women more often had impairment in the domains of attention, executive functioning, and language, whereas men more often had impairment in verbal memory. In addition, the MMSE had a higher sensitivity in women compared with men, but the specificity was lower in women compared with men.

Few previous studies reported on differences between women and men in PSCI. Some studies reported that women have a higher risk of PSCI, but others reported no relevant differences.^6–9^ Most previous studies used cognitive screening tests with general cutoffs to establish PSCI, in contrast to the current study that used an NTB and norm-referenced data to define cognitive impairment. Only one previous study used multidomain neuropsychological testing. In the CogFast Nigeria study (n=143),^9^ female sex was associated with an increased risk of PSCI (OR, 2.27 [95% CI, 1.15–4.45]). Two previous studies (n=302, n=342), using MoCA to identify PSCI, also have shown that female sex was associated with an increased risk of cognitive impairment (OR, 1.60 [95% CI, 1.01–2.57] and OR, 1.72 [95% CI, 1.05–2.80]).^6,8^ Compared with the current study, these cohorts were modest in size and included both ischemic and hemorrhagic stroke. Another previous study that used the modified MMSE (n=1227; included both ischemic and hemorrhagic stroke) did not find sex differences in the risk of PSCI.^7^

The current study further shows that the cognitive profile of PSCI differs by sex, an understudied topic. In a Korean cohort (n=141) of stroke patients with mild PSCI, men had an increased risk of impairment in verbal memory (OR, 3.07 [95% CI, 1.12–8.42]) compared with women.^10^ This is in line with our findings, although they did not report on other cognitive domains. In general, women at all ages perform better on verbal memory tasks,^26^ and men perform better on visuospatial tasks.^27^ In patients with Alzheimer dementia, women have better verbal memory performance than men,^11,28^ but this is less clear for visuospatial tasks.^11^ Application of sex-specific cut scores for defining verbal memory impairment has previously been shown to improve diagnostic accuracy in both sexes and avoids false diagnoses in men.^27^ In the current study, we have applied norm-referenced data, adjusted for sex when applicable. Our findings show that, in line with Alzheimer dementia, women are also less likely to have verbal memory impairment after ischemic stroke. Future research will need to investigate potential differences in cognitive trajectories by sex in PSCI.

There are no previous studies on sex differences in the performance of cognitive screening tools for PSCI. Our results show that the MMSE has a higher sensitivity for detecting PSCI in women than men, but the specificity was lower in women than men. The sensitivity and specificity of MoCA was comparable between the sexes. However, the sample size of patients with NTB and MoCA was small (women n=83, men n=173) compared with the subgroup with NTB and MMSE (women n=722, men n=1091). Also in the 3 additional cohorts without NTB but with an available MoCA score (n=607; Table S7) more women (84%) than men (66%) had a MoCA score below the cutoff (<26). This pattern differs from our main results in which, based on NTB, PSCI occurrence was equal in women and men. This suggests that MoCA might perform differently by sex in poststroke patients. Overall, both MMSE and MoCA performed modestly as stand-alone screening instruments, thus caution is warranted when cognitive screening tools are used to identify PSCI. In clinical practice and research, it could cause over- or underestimating cognitive impairment in one sex.

The sex differences in cognitive profile of PSCI have several potential consequences. First, different profiles of PSCI by sex likely lead to different experienced disabilities when trying to resume activities of daily life.^29^ In clinical practice, consideration of the impaired and preserved cognitive abilities of a patient can help tailor communication and rehabilitation strategies. Second, due to the difference in cognitive profiles, detection of PSCI might differ between sexes, depending on the cognitive screening test or NTB used. Lastly, the different performance of cognitive screening tests by sex might be related to sex differences in cognitive profile. The MMSE has a larger focus on verbal abilities than MoCA, whereas MoCA contains more items testing executive and visuoconstructive function and covers more cognitive domains than MMSE. Although women in general perform better on verbal memory tasks, this seems only to be reflected by MoCA and not by the MMSE.^30^ This may be due to differences in difficulty between tasks on each test, namely verbal recall of 3 words in the MMSE compared with 5 words in the MoCA).^30^ In the current study, we found that women are less likely to have verbal memory impairment. This could explain previous conflicting findings, where a study^7^ that used the modified MMSE did not find sex differences, while 2 studies^6,8^ that used MoCA reported women had a higher risk of PSCI. Overall, the tested cognitive domains influence the chance of detecting PSCI in women and men. A multidomain cognitive assessment with norm-referenced data is less likely to underestimate the occurrence of PSCI in either sex.

Strengths of the current study are the large sample size and availability of multidomain cognitive data, that allowed us to report on cognitive profiles. Our definition of PSCI, a binary measure that covered impairment in any cognitive domain, is in line with internationally established criteria^20^ and reflects the diagnosis of cognitive impairment in clinical practice. Although neuropsychological test batteries differed between cohorts, our PSCI definition did allow us to cover a broad range of cognitive profiles and deficits. Secondly, we were able to use norm-referenced data to define impairment. Performance on cognitive tests is known to differ by geographic region and can be influenced by age, educational level, and sometimes sex. Applying normative data in research cohorts improves diagnostic accuracy in both women and men.^31^ Third, we included multicenter data that enabled a much larger sample size than individual studies to date. However, this inherently resulted in heterogeneity.

Some potential limitations must also be noted. First, information on prestroke cognitive status was not available for a substantial number of cohorts, therefore we could not take this into account in our analyses. Second, women are known to be underrepresented in stroke studies, possibly introducing bias. Also in the current pooled data, more men (62%) than women (38%) were included. Thirdly, MoCA scores were available only for a small sample to calculate sensitivity and specificity, and therefore, those results should be interpreted with caution. Fourthly, we did not account for prestroke brain changes such as features of small vessel disease that are known to increase PSCI and differ between men and women: this can be addressed in future research. Finally, our pooled sample only includes White and Asian patients, thus generalizability to other ethnicities remains undetermined.

## ARTICLE INFORMATION

### Sources of Funding

Dr Exalto is supported by Alzheimer Nederland WE.03-2019-15 and Netherlands CardioVascular Research Initiative: the Dutch Heart Foundation (CVON 2018-28 & 2012-06). The Meta-VCI Map consortium is supported by Vici Grant 918.16.616 from The Netherlands Organisation for Health Research and Development (ZonMw) to Dr Biessels. Harmonization analyses were supported by a Rudolf Magnus Young Talent Fellowship from the University Medical Center Utrecht Brain Center to Dr Biesbroek. The CASPER cohort was supported by Maastricht University, Health Foundation Limburg, and Stichting Adriana van Rinsum-Ponsen. The CROMIS-2 cohort was funded by the UK Stroke Association and the British Heart Foundation (grant number TSA BHF 2009/01). The CU-STRIDE cohort was supported by the Health and Health Services Research Fund of the Food and Health Bureau of the Government of Hong Kong (grant number 0708041), the Lui Che Woo Institute of Innovative Medicine, and Therese Pei Fong Chow Research Center for Prevention of Dementia. The GRECogVASC cohort was funded by Amiens University Hospital and by a grant from the French Ministry of Health (grant number DGOS R1/2013/144). The MSS-2 cohort is funded by the Wellcome Trust (grant number WT088134/Z/09/A to Dr Wardlaw) and the Row Fogo Charitable Trust. The PROCRAS cohort was funded via ZonMW as part of the TopZorg project in 2015 (grant number 842003011). The CODECS cohort (ongoing) is supported by a grant from Stichting Coolsingel (grant number 514). The Bundang VCI and Hallym VCI cohort groups do not wish to report any relevant funding sources. At the time of contribution, Dr Hamilton was funded by the College of Medicine and Veterinary Medicine at the University of Edinburgh and was supported by the Wellcome Trust through the Translational Neuroscience PhD program at the University of Edinburgh.

### Disclosures

Dr Aben reports grants from ZonMW (grant number 842003011). Dr Chen reports grants from the National Medical Research Council (NMRC) of Singapore and National University of Singapore. Dr Godefroy reports grants from Amiens University Hospital and the French Ministry of Health, during the last 5 years Dr Godefroy has served on scientific advisory boards and as a speaker for Novartis, CSL-Behring, Biogen, Genzyme, Lilly, Bristol-Myers Squibb, Boehringer-Ingelheim, Covidien, Teva Santé, and Astra Zeneca. Dr Hamilton is supported by the Medical Research Council (MC_UU_00022/2) and the Scottish Chief Scientist Office (SPHSU17). Dr Köhler reports grants from Nederlandse Organisatie voor Wetenschappelijk Onderzoek and grants from ZonMw. Dr Wardlaw reports grants from the Wellcome Trust, Row Fogo Charitable Trust, and the UK Medical Research Council. Dr Werring has received: grant funding from the Stroke Association and British Heart Foundation; speaking honoraria from Bayer; speaking and chairing honoraria from Alexion and NovoNordisk; and consultancy fees from Bayer and NovoNordisk. Dr Biessels reports grants from ZonMW and Hartstichting. The other authors report no conflicts.

### Supplemental Material

Checklist

Tables S1–S8

Figures S1–S3

## Supplementary Material

## References

[1] Pollock A St George B Fenton M Firkins L. Top ten research priorities relating to life after stroke. Lancet Neurol. 2012;11:209. doi: 10.1016/S1474-4422(12)70029-7 22341029 10.1016/S1474-4422(12)70029-7

[2] Pendlebury ST Rothwell PM; Oxford Vascular Study. Incidence and prevalence of dementia associated with transient ischaemic attack and stroke: analysis of the population-based Oxford Vascular Study. Lancet Neurol. 2019;18:248–258. doi: 10.1016/S1474-4422(18)30442-3 30784556 10.1016/S1474-4422(18)30442-3 PMC6390174

[3] Barbay M Diouf M Roussel M Godefroy O. Systematic review and meta-analysis of prevalence in post-stroke neurocognitive disorders in hospital-based studies. Dement Geriatr Cogn Disord. 2019;46:322–334. doi: 10.1159/000492920 10.1159/000492920 30504699

[4] Stolwyk RJ Mihaljcic T Wong DK Chapman JE Rogers JM. Poststroke cognitive impairment negatively impacts activity and participation outcomes: a systematic review and meta-analysis. Stroke. 2021;52:748–760. doi: 10.1161/STROKEAHA.120.032215 33493048 10.1161/STROKEAHA.120.032215

[5] Gall S Phan H Madsen TE Reeves M Rist P Jimenez M Lichtman J Dong L Lisabeth LD. Focused update of sex differences in patient reported outcome measures after stroke. Stroke. 2018;49:531–535. doi: 10.1161/STROKEAHA.117.018417 29438087 10.1161/STROKEAHA.117.018417

[6] Mellon L Brewer L Hall P Horgan F Williams D Hickey A McGee H Shelley E Kelly P Dolan E. Cognitive impairment six months after ischaemic stroke: a profile from the ASPIRE-S study. BMC Neurol. 2015;15:1–9. 25879880 10.1186/s12883-015-0288-2 PMC4359388

[7] Dong L Briceno E Morgenstern LB Lisabeth LD. Poststroke cognitive outcomes: sex differences and contributing factors. J Am Heart Assoc. 2020;9:e016683. doi: 10.1161/JAHA.120.016683 32633589 10.1161/JAHA.120.016683 PMC7660722

[8] Swardfager W MacIntosh BJ. Depression, type 2 diabetes, and poststroke cognitive impairment. Neurorehabil Neural Repair. 2017;31:48–55. doi: 10.1177/1545968316656054 27364648 10.1177/1545968316656054

[9] Akinyemi RO Allan L Owolabi MO Akinyemi JO Ogbole G Ajani A Firbank M Ogunniyi A Kalaria RN. Profile and determinants of vascular cognitive impairment in African stroke survivors: the CogFAST Nigeria Study. J Neurol Sci. 2014;346:241–249. doi: 10.1016/j.jns.2014.08.042 25238666 10.1016/j.jns.2014.08.042

[10] Cho SJ Yu KH Oh MS Jung S Lee JH Koh IS Bae HJ Kang Y Lee BC; Korean-Vascular Cognitive Impairment Harmonization Standards Study Group. Post-stroke memory impairment among patients with vascular mild cognitive impairment. BMC Neurol. 2014;14:244. doi: 10.1186/s12883-014-0244-6 25927318 10.1186/s12883-014-0244-6 PMC4300833

[11] Cox JL. Sex differences in cognitive impairment in Alzheimer’s disease. World Psychiatry. 2016;6:54–56. 10.5498/wjp.v6.i1.54 PMC4804268 27014598

[12] Weaver NA Zhao L Biesbroek JM Kuijf HJ Aben HP Bae HJ Caballero MAA Chappell FM Chen CPLH Dichgans M; Meta VCI Map consortium. The Meta VCI Map consortium for meta-analyses on strategic lesion locations for vascular cognitive impairment using lesion-symptom mapping: design and multicenter pilot study. Alzheimer’s Dement. 2019;11:310–326. doi: 10.1016/j.dadm.2019.02.007 10.1016/j.dadm.2019.02.007 PMC6465616 31011619

[13] Weaver NA Kuijf HJ Aben HP Abrigo J Bae H Barbay M Best JG Bordet R Chappell FM Kuchcinski G. Articles Strategic infarct locations for post-stroke cognitive impairment: a pooled analysis of individual patient data from 12 acute ischaemic stroke cohorts. Lancet Neurol. 2021;4422:1–12. 10.1016/S1474-4422(21)00060-0 33901427

[14] Lo JW Crawford JD Desmond DW Godefroy O Jokinen H Mahinrad S Bae HJ Lim JS Köhler S Douven E; Stroke and Cognition (STROKOG) Collaboration. Profile of and risk factors for poststroke cognitive impairment in diverse ethnoregional groups. Neurology. 2019;93:e2257–e2271. doi: 10.1212/WNL.0000000000008612 31712368 10.1212/WNL.0000000000008612 PMC6937495

[15] Brott T Adams HP Olinger CP Marler JR Barsan WG Biller J Spilker J Holleran R Eberle R Hertzberg V. Measurements of acute cerebral infarction: a clinical examination scale. Stroke. 1989;20:864–870. doi: 10.1161/01.str.20.7.864 2749846 10.1161/01.str.20.7.864

[16] Jorm AF. A short form of the Informant Questionnaire on Cognitive Decline in the Elderly (IQCODE): development and cross-validation. Psychol Med. 1994;24:145–153. doi: 10.1017/s003329170002691x 8208879 10.1017/s003329170002691x

[17] Wardlaw JM Smith EE Biessels GJ Cordonnier C Fazekas F Frayne R Lindley RI O’Brien JT Barkhof F Benavente OR; STandards for ReportIng Vascular changes on nEuroimaging (STRIVE v1). Neuroimaging standards for research into small vessel disease and its contribution to ageing and neurodegeneration. Lancet Neurol. 2013;12:822–838. doi: 10.1016/S1474-4422(13)70124-8 23867200 10.1016/S1474-4422(13)70124-8 PMC3714437

[18] Weaver NA Kancheva AK Lim J Biesbroek JM Mc I Wajer H Kang Y Kim BJ Kuijf HJ Lee B. Post-stroke cognitive impairment on the Mini-Mental State Examination primarily relates to left middle cerebral artery infarcts. Int J Stroke. 2021;0:1–9. 10.1177/1747493020984552 PMC8554493 33472574

[19] Lezak MD. Neuropsychological assessment. 2004;4th ed.

[20] Sachdev P Kalaria R O’Brien J Skoog I Alladi S Black SE Blacker D Blazer DG Chen C Chui H; Internationlal Society for Vascular Behavioral and Cognitive Disorders. Diagnostic criteria for vascular cognitive disorders: a VASCOG statement. Alzheimer Dis Assoc Disord. 2014;28:206–218. doi: 10.1097/WAD.0000000000000034 24632990 10.1097/WAD.0000000000000034 PMC4139434

[21] Folstein MF Folstein SE McHugh PR. “Mini-mental state.” A practical method for grading the cognitive state of patients for the clinician. J Psychiatr Res. 1975;12:189–198. doi: 10.1016/0022-3956(75)90026-6 1202204 10.1016/0022-3956(75)90026-6

[22] Nasreddine ZS Phillips NA Bédirian V Charbonneau S Whitehead V Collin I Cummings JL Chertkow H. The Montreal Cognitive Assessment, MoCA: a brief screening tool for mild cognitive impairment. J Am Geriatr Soc. 2005;53:695–699. doi: 10.1111/j.1532-5415.2005.53221.x 15817019 10.1111/j.1532-5415.2005.53221.x

[23] The Cochrane Handbook for Systematic Reviews of Diagnostic Test Accuracy was used for the analysis. https://methods.cochrane.org/sdt/handbook-dta-reviews. 10.1002/14651858.ED000163 PMC10408284 37470764

[24] Leeflang MMG Rutjes AWS Reitsma JB Hooft L Bossuyt PMM. Variation of a test’s sensitivity and specificity with disease prevalence. CMAJ. 2013;185:E537–E544. doi: 10.1503/cmaj.121286 23798453 10.1503/cmaj.121286 PMC3735771

[25] von Elm E Altman DG Egger M Pocock SJ Gøtzsche PC Vandenbroucke JP; STROBE Initiative. The Strengthening the Reporting of Observational Studies in Epidemiology (STROBE) statement: guidelines for reporting observational studies. J Clin Epidemiol. 2008;61:344–349. doi: 10.1016/j.jclinepi.2007.11.008 18313558 10.1016/j.jclinepi.2007.11.008

[26] Sundermann EE Maki PM Rubin LH Lipton RB Landau S Biegon A; Alzheimer's Disease Neuroimaging Initiative. Female advantage in verbal memory: evidence of sex-specific cognitive reserve. Neurology. 2016;87:1916–1924. doi: 10.1212/WNL.0000000000003288 27708128 10.1212/WNL.0000000000003288 PMC5100712

[27] Mielke MM Vemuri P Rocca WA. Clinical epidemiology of Alzheimer’s disease: Assessing sex and gender differences. Clin Epidemiol. 2014;6:37–48. doi: 10.2147/CLEP.S37929 24470773 10.2147/CLEP.S37929 PMC3891487

[28] Tensil M Hessler JB Gutsmiedl M Riedl L Grimmer T Diehl-Schmid J. Sex Differences in neuropsychological test performance in Alzheimer’s disease and the influence of the ApoE genotype. Alzheimer Dis Assoc Disord. 2018;32:145–149. doi: 10.1097/wad.0000000000000229 29189302 10.1097/WAD.0000000000000229

[29] Aam S Einstad MS Munthe-Kaas R Lydersen S Ihle-Hansen H Knapskog AB Ellekjær H Seljeseth Y Saltvedt I. Post-stroke cognitive impairment—impact of follow-up time and stroke subtype on severity and cognitive profile: the nor-COAST study. Front Neurol. 2020;11:699. doi: 10.3389/fneur.2020.00699 32765406 10.3389/fneur.2020.00699 PMC7379332

[30] Pendlebury ST Cuthbertson FC Welch SJV Mehta Z Rothwell PM. Underestimation of cognitive impairment by mini-mental state examination versus the montreal cognitive assessment in patients with transient ischemic attack and stroke: a population-based study. Stroke. 2010;41:1290–1293. doi: 10.1161/STROKEAHA.110.579888 20378863 10.1161/STROKEAHA.110.579888

[31] Sundermann EE Maki P Biegon A Lipton RB Mielke MM Machulda M Bondi MW; Alzheimer's Disease Neuroimaging Initiative. Sex-specific norms for verbal memory tests may improve diagnostic accuracy of amnestic MCI. Neurology. 2019;93:e1881–e1889. doi: 10.1212/WNL.0000000000008467 31597708 10.1212/WNL.0000000000008467 PMC6946472

