# Supplementary material for: Sex Differences in Poststroke Cognitive Impairment: A Multicenter Study in 2343 Patients With Acute Ischemic Stroke
Source: Stroke. 2023 Aug 8;54(9):2296–303. doi: 10.1161/STROKEAHA.123.042507 (PMC10453354; doi:10.1161/STROKEAHA.123.042507)
Supplement: Supplementary file 1 [file str-54-2296-s001.pdf]

Supplements

Methods

Table S1. Details on participating cohorts

Study	Country	Single or multicenter	Period of inclusion	Inclusion criteria	Exclusion criteria
Bundang VCI	Republic of Korea	Single	2007 - 2018	• Ischemic stroke, hospitalized within 1 week of onset<br>• acute ischemic lesions on diffusion-weighted imaging<br>• Informed consent obtained	• Severe concomitant medical or neurological conditions (persistent impairment of consciousness or visual impairment)<br>• Severe dysphasia<br>• Death within 2 weeks of stroke onset
CASPER	Netherlands	Multi	2013 – 2016	• Ischemic or hemorrhagic stroke<br>• MMSE score ≥ 15<br>• Written informed consent<br>• Sufficient knowledge of the Dutch language	• Subarachnoid hemorrhage, traumatic hemorrhage, primary intraventricular hemorrhage and transient ischemic attack<br>• Age < 40 years<br>• Severe aphasia<br>• Evidence for pre-stroke dementia (based on clinical diagnosis or IQ-CODE) in the 5 years prior to the stroke<br>• Other existing psychiatric and neurological diagnoses that are known to affect cognition (Parkinson's disease, bipolar disorder, epilepsy, schizophrenia, or substance abuse)
COAST	Singapore	Multi	2009 – 2017	• Age ≥21 years<br>• Acute ischaemic stroke or TIA with onset within the preceding 14 days<br>• Stable clinical and neurological status within the preceding 24 hours<br>• Written consent obtained from patient or legally acceptable representative	• Significant aphasia and/or dysarthria that impedes performance of cognitive assessment<br>• Major and active psychiatric illness<br>• Acute delirium<br>• Pre-existing dementia<br>• Major physical disability with modified Rankin Scale score >4
CODECS	Netherlands	Single	2015 – 2019	• Age ≥18 years<br>• Isolated cerebellar stroke	• Significant aphasia or severe dysarthria<br>• Prior cognitive impairment
GRECogVASC	France	Multi	2010 – 2015	• Age between 40 and 80 years<br>• Hospitalized for acute (<30 days) cerebral infarct or hemorrhage with initial positive imaging<br>• No previously diagnosed conditions affecting cognition (except for previous stroke)<br>• French-speaking<br>• Reliable informant, agreeing to participate in the study	• Mental retardation, illiteracy<br>• Known dementia<br>• Schizophrenia or psychosis or history of psychiatric illness requiring a stay > 2 days in a psychiatry unit<br>• Persistent disturbance of consciousness<br>• Contraindication to MRI<br>• For the present analysis: subset of 316 patients with infarct and MR assessment in Amiens center

Hallym VCI	Republic of Korea	Single	2007 - 2018	• Ischemic stroke, hospitalized within 1 week of onset<br>• Acute ischemic lesions on diffusion-weighted imaging<br>• Informed consent obtained	• Severe concomitant medical or neurological conditions (persistent impairment of consciousness or visual impairment)<br>• Severe dysphasia<br>• Death within 2 weeks of stroke onset
PROCRAS	Netherlands	Single	2016 – 2018	• Clinical diagnosis of ischemic stroke<br>• Age ≥50 years	• Pre-stroke dementia: Known diagnosis of dementia or Informant Questionnaire on Cognitive Decline in the Elderly (IQCODE) ≥3.6<br>• Life expectancy <1 year<br>• Severe stroke expected to require long-term nursing care facilities<br>• History of major neurological disease interfering with cognitive functioning<br>• Pre-stroke dependence in activities of daily living (Barthel Index<18)<br>• Insufficient command of the Dutch language to participate and understand questionnaires<br>• Impossibility to participate in a neuropsychological assessment<br>• An absolute contraindication to undergo an MRI scan of the brain
STROKDEM	France	Multi	2011 – 2018	• Age >40 years<br>• Hemispheric stroke<br>• Stroke dating from less 72h<br>• IQCODE < 64<br>• Patient (or his family) given an informed consent	• Malformed cerebral hemorrhage, traumatic cerebral hemorrhage, pure meningeal or intraventricular hemorrhage<br>• Contraindications to MRI<br>• Insufficient mastery of the French language<br>• No informed consent
USCOG	Netherlands	Single	2005 - 2012	• First-ever ischemic stroke<br>• Brain infarction on follow-up CT or MRI	• Pre-existent neurologic conditions that might interfere with cognition: history of cognitive impairment, traumatic brain injury, brain tumor, epilepsy, multiple sclerosis, moyamoya disease, or severe cerebral small vessel disease (i.e. Fazekas grade 3)

More details can be found in previous publication, references listed in main text [12, 13]

Table S2. Overview of cohorts and available neuropsychological data per cohort

Study	N=	Cognitive screening	Attention & Executive Functioning	Information processing speed	Language	Verbal memory	Visuospatial memory	Visuoperception and -construction
Bundang VCI	753	MMSE	1. TMT B<br>2. Phonemic fluency	1. TMT A<br>2. Digit Symbol Coding	1. Boston Naming Test<br>2. Semantic fluency – animals	Seoul Verbal Learning Test:<br>1. Immediate recall<br>2. Delayed recall<br>3. Recognition	N/A	1. Rey Complex Figure Test: copy
CASPER	104	MMSE	1. TMT B<br>2. Digit span forward<br>3. Digit span backward	1. TMT A	1. Semantic fluency – animals	Rey Auditory Verbal Learning Test<br>1. Immediate recall<br>2. Delayed recall<br>3. Recognition	N/A	N/A
COAST	74	MoCA	1. Visual memory span forward<br>2. Visual memory span backward<br>3. Auditory Detection test<br>4. Digit cancellation task<br>5. Maze task<br>6. Digit span forward<br>7. Digit span backward	1. Symbol Digit Modalities Test	1. Boston Naming Test<br>2. Semantic fluency – animals	Word-List Recall<br>1. Immediate<br>2. Delayed<br>3. Recognition<br><br>Story Recall<br>4. Immediate<br>5. Delayed	Picture Recall<br>1. Immediate<br>2. Delayed<br>3. Recognition<br><br>Visual Reproduction<br>4. Immediate<br>5. Delayed<br>6. Recognition	1. Clock-drawing<br>2. Block design<br>3. Visual reproduction copy
CODECS	27	MoCA	1. TMT B<br>2. Phonemic fluency<br>3. Stroop	1. TMT A	4. Semantic fluency – animals	N/A	N/A	N/A
CROMIS-2	97	MoCA	N/A	N/A	N/A	N/A	N/A	N/A
CU-STRIDE	410	MoCA<br>MMSE	N/A	N/A	N/A	N/A	N/A	N/A
GRECogVASC	316	MMSE	1. TMT B<br>2. Phonemic fluency	1. TMT A<br>2. Digit Symbol Coding	1. Boston Naming Test<br>2. Semantic fluency – animals	Free and Cued Selective Reminding Test<br>1. Immediate<br>2. Delayed<br>3. Sum 3 total recall<br>4. Recognition	N/A	1. Rey Complex Figure Test: copy
Hallym VCI	641	MMSE	1. TMT B<br>2. Phonemic fluency	1. TMT A<br>2. Digit Symbol Coding	1. Boston Naming Test<br>2. Semantic fluency – animals	Seoul Verbal Learning Test<br>1. Immediate recall<br>2. Delayed recall<br>3. Recognition	1. Rey Complex Figure Test: delayed recall	1. Rey Complex Figure Test: copy

Mild Stroke Study 2	100	MoCA	N/A	N/A	N/A	N/A	N/A	N/A
PROCRAS	177	MoCA	1. TMT B<br>2. Phonemic fluency<br>3. Hayling test<br>4. Reaction time test, Vienna Test System S3<br>5. Digit span forward<br>6. Digit span backward	1. TMT A<br>2. Symbol Digit Modalities Test<br>3. Reaction time test, Vienna Test System S1<br>4. Reaction time test, Vienna Test System S2	1. Boston naming Test<br>2. Semantic fluency – animals	Rey Auditory Verbal Learning Test<br>1. Immediate recall<br>2. Delayed recall	N/A	N/A
STROKDEM	138	N/A	1. TMT B<br>2. Phonemic fluency<br>3. Stroop	1. TMT A<br>2. Digit Symbol Coding	1. Semantic fluency – animals<br>2. D080 – picture naming	Free and Cued Selective Reminding Test<br>1. Immediate recall<br>2. Delayed free recall	1. Rey Complex Figure Test: immediate recall	1. Rey Complex Figure Test: copy<br><br>Visual object and space perception battery<br>2. Incomplete letters<br>3. Number location
USCOG	113	N/A	1. Phonemic fluency<br>2. BADS zoo test<br>3. Digit span forward<br>4. Digit span backward	N/A	1. Boston naming Test<br>2. Semantic fluency – animals<br>3. Token test	Rey Auditory Verbal Learning Test<br>1. Immediate recall<br>2. Delayed recall<br>3. Recognition	1. Rey Complex Figure Test: delayed recall	1. Rey Complex Figure Test: copy<br>2. Judgment of Line Orientation

Categorization of neuropsychological tests was based on previous work by Lezak.[19] The length of the numbered list indicates the maximum number of tests available in a cohort. Note that for some tests, individual subscores were separately included (e.g. immediate recall, delayed recall and recognition counting as 3 separate components in the verbal memory domain). For determining presence or absence of post-stroke cognitive impairment, availability of tests was determined on a per-subject basis. Normative data for each cohort is shown in Supplementary Table 3.

Abbreviations: BADS, Behavioral Assessment of the Dysexecutive Syndrome; MoCA, Montreal Cognitive Assessment; N/A, not applicable or not available; TMT, Trail Making Test.

Table S3. Normative data for detailed neuropsychological assessment

Study name	N of control group	Population
**Studies that recruited their own control group**		
GRECogVASC	1003	General population not presenting any condition known to impair cognitive abilities stratified according to age and schooling levels.
**Studies that provided control group data from a separate local study**		
COAST	279	A subset without cognitive impairment from the EDIS study. EDIS is a Singapore study with participants drawn from the Singapore Epidemiology of Eye Disease study, a multiethnic population-based study among persons aged 40–85 years, which included Chinese, Malays and Indians.
**Studies that calculated standardised scores based on published local norms or studies**		
Bundang VCI and Hallym VCI	Varied	Age, sex and education matched community dwelling elderly.S3
CASPER	1823	Maastricht Aging Study. Participants were drawn from a patient register of collaborating general practitioners.
CODECS	Varied	Dutch population-based normative data adjusted for age, sex and level of education. Published in 2012 on website of The Dutch Association of Psychologists (https://www.psynip.nl/wp-content/uploads/2016/07/Handleiding-normen-Np-tests-2012.pdf).
PROCRAS	Varied	• Phonemic fluency, semantic fluency, TMT A and B, Rey Auditory Verbal Learning Test: Dutch population-based normative data adjusted for age, sex and level of education. Published in 2012 on website of The Dutch Association of Psychologists (https://www.psynip.nl/wp-content/uploads/2016/07/Handleiding-normen-Np-tests-2012.pdf).<br>• Boston Naming Test: Heesbeen 2002 [16], adjusted for age and education.<br>• Digit Span forward/backward, Vienna Test System, SDMT: Dutch normative data from official manuals.<br>• Hayling: international normative data from official manual.
STROKDEM	Varied	• Verbal Fluency and Trail Making Test: Based on the work of Roussel and Godefroy, z-scores were calculated by age and education group. Note that these scores were not adjusted for sex.<br>• Rey Complex Figure Test: Expected scores for copy and immediate recall were computed using equations from Tremblay et al. adjusted for sex, age, and education. Then, z-scores were computed from the expected scores.
USCOG	Varied	• Phonemic fluency, semantic fluency, and Rey Auditory Verbal Learning Test: Dutch population-based normative data adjusted for age, sex and level of education. Published in 2012 on website of The Dutch Association of Psychologists (https://www.psynip.nl/wp-content/uploads/2016/07/Handleiding-normen-Np-tests-2012.pdf).<br>• Boston Naming Test: Heesbeen 2002 [16], adjusted for age and education.<br>• Digit Span forward/backward and Token Test: Dutch normative data from official manuals, adjusted for age.<br>• Token Test: Dutch normative data from official manuals, adjusted for age and IQ.<br>• Rey Complex Figure Test: copy: normative data adjusted for age and education.<br>• Judgment of Line Orientation: normative data adjusted for age and education.

Figure S1. Sex differences in risk of PSCI per cohort.

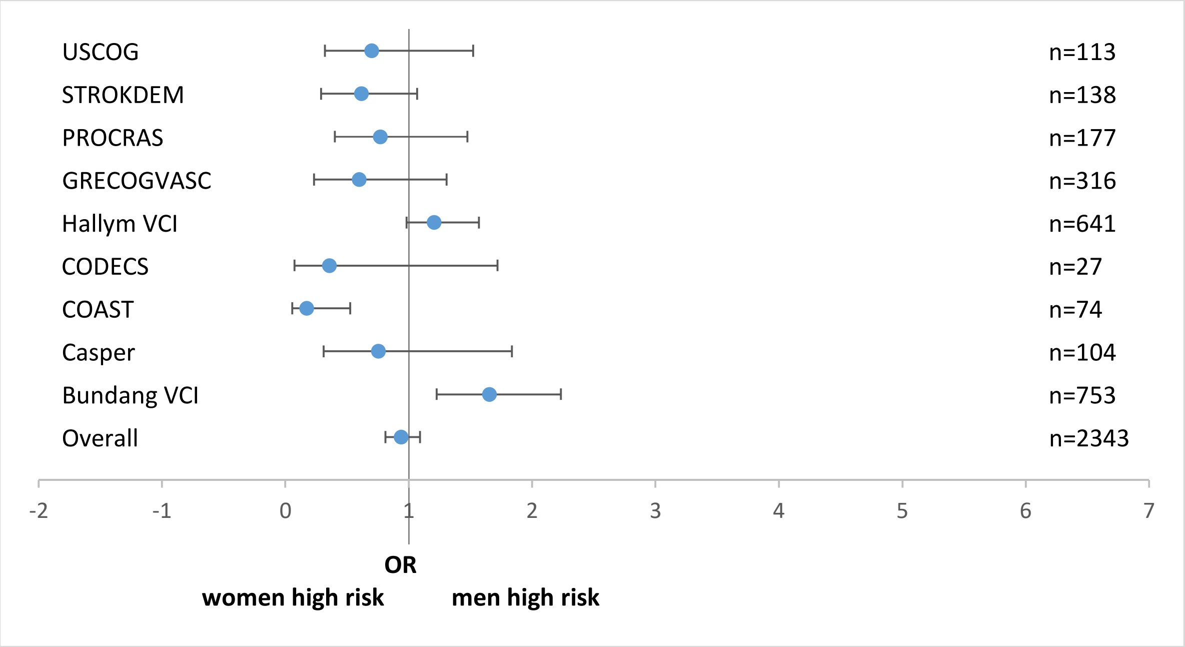

Details about the different cohorts like in and exclusion criteria can be found in supplementary table 1.

Results

Table S4. Baseline characteristics of individual cohorts

Characteristics	Main analysis (cohorts with NTB)									Additional cohorts (without NTB)		
	Bundang VCI (n = 753)	CASPER (n = 104)	COAST (n = 74)	CODECS (n = 27)	GRECogVAS C (n = 316)	Hallym VCI (n = 641)	PROCRAS (n = 177)	STROKDEM (n = 138)	USCOG (n = 113)	CROMIS-2 (n = 97)	CU-STRIDE (n = 410)	MSS-II (n = 100)
**Demographic characteristics**												
Age in years, mean (SD)	69.8 ±10.8	64.1 ±10.8	58.4 ±10.5	59.2 ±16.5	63.7 ±10.6	65.1 ±11.9	69.6 ±9.4	64.9 ±12.1	60.0 ±14.9	73.7 ±9.2	68.6 ±10.4	65.7 ±11.5
Women, n (%)	306 (41)	27 (26)	21 (28)	12 (44)	121 (38)	268 (42)	58 (33)	53 (38)	47 (42)	43 (44)	163 (40)	38 (38)
Education level (STROKOG)												
- Less than high school	402 (53)	42 (40)	52 (70)	8 (30)	233 (74)	351 (55)	79 (45)	86 (62)	44 (39)	6 (6)	381 (93)	19 (19)
- High school completion	146 (19)	19 (18)	14 (19)	4 (15)	30 (10)	154 (24)	53 (30)	16 (12)	27 (24)	78 (80)	5 (1)	62 (62)
- Technical/college diploma	37 (5)	35 (34)	7 (10)	5 (19)	35 (11)	31 (5)	39 (22)	11 (8)	26 (23)	N/A	8 (2)	N/A
- University or higher	168 (22)	8 (8)	1 (1)	10 (37)	18 (6)	105 (16)	6 (3)	25 (18)	16 (14)	13 (13)	16 (4)	19 (19)
**Clinical characteristics**												
NIHSS at baseline, media [IQR]	3 [2-5]	N/A	3 [1-7]	0 [0-0]	3 [1-6]	2 [1-4]**	3[2-4]	0 [0-1]	N/A	3 [2-6]	4 [2-6]	1 [0-2]
IQCODE, media [IQR]	3.3 [4.1-3.7]**	3.1 [3.0-3.3]**	3.0 [3.0-3.1]*	N/A	0% impaired	3.1 [3.0-3.3]***	3.0 [3.0-3.1]**	3.0[3.0-3.1]	N/A	3.0 [3.0-3.3]	N/A	N/A
**Medical history, n (%)**												
Hypertension	175 (23)	80 (77)	56 (76)	15 (56)	185 (59)	385 (60)*	132 (75)	76 (55)	20 (39)***	54 (56)*	305 (74)	76 (76)
Hyperlipidemia	201 (27)	88 (85)	59 (80)	9 (33)	136 (43)	246 (39)**	167 (94)	60 (44)	12 (23)***	48 (50)*	242 (59)	64 (64)
Diabetes mellitus	246 (33)	14 (14)	36 (49)	7 (26)	66 (21)	195 (31)*	52 (29)	18 (13)	6 (11)***	11 (12)*	151 (37)	12 (13)*
History of stroke	104 (14)	5 (5)	10 (13.5)[a]	0 (0)	22 (7)	85 (14)**	23 (13)	12 (9)	0 (0)	6 (6)*	50 (12)	10 (10)
**Brain imaging**												
Normalized acute infarct volume in ml, median [IQR]	3.8 [1.2-16.5]	3.4 [0.9-13.2]	6.8 [2.0-32.6]	10.3 [1.1-25.2]	1.3 [0.3-5.7]	2.0 [0.9-11.3]	4.4 [1.3-21]	1.6 [0.6-8.6]	19.6 [3.5-51.9]	4.5 [1.5-16.1]	2.3 [0.9-12.9]	2.7 [1.0-14.1]
Imaging timing, n days after event, median [IQR]	5 [4-6]	87 [81-99]	2 [1-4]	34 ([5-98]**	178 [161-186]	1 [1-2]	33 [27-40]	3 [3-3]	5 [3-8]	5 [3-9]	1 [0-2]	4 [1-9]
**Cognitive testing**												
Timing cognitive assessment, n days after event, median [IQR]	104 [10-170]	87 [81-99]	121 [105-152]	90 [NA]	178 [161-186]	98 [90-105]	35 [29-40]	189 [178-199]	6 [4-9]	4 [2-9]	154 [129-176]	142 [53-383]

* Missing in <1%; ** Missing in 1-10%; *** Missing in >10%

[a] Combined variable for stroke and/or TIA

Abbreviations: IQR, interquartile range; MoCA, Montreal Cognitive Assessment; SD, standard deviation; TIA, transient ischemic attack

Table S5. Timing of cognitive testing post-stroke.

	0-3 months	3-6 months	6-9 months	9-12 months
**Women, n (%)**	345 (38%)	403 (44%)	142 (16%)	20 (2%)
**Men, n (%)**	558 (39%)	614 (43%)	223 (16%)	35 (2%)

Figure S2. Sex differences in risk of impairment in cognitive domains stratified by demographics and stroke type.

a. Attention and Executive Functioning

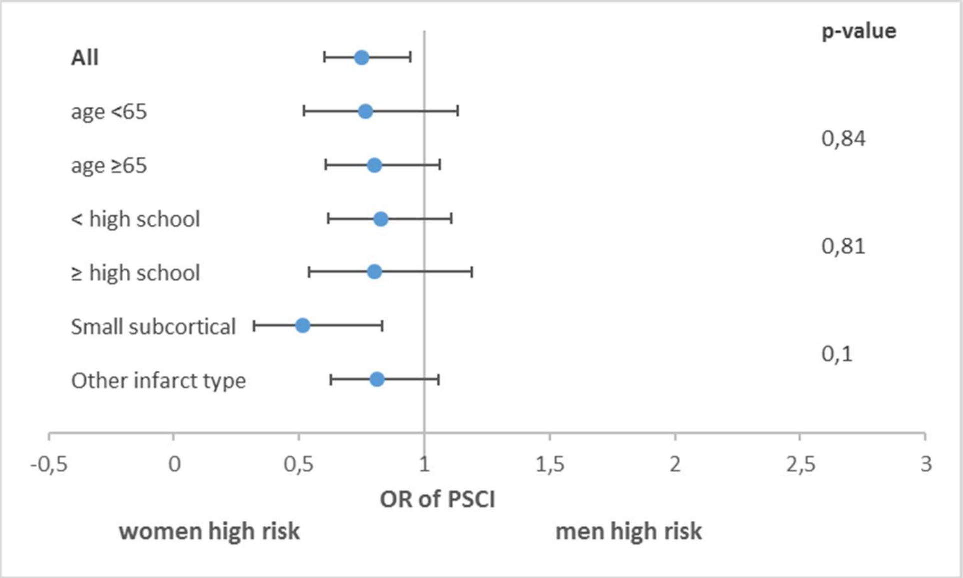

N=2195; missing 148 (6%).
Logistic regression analyses were performed to obtain OR for PSCI; females denoted the reference group.
The p-values depict the p-value of the interaction term stratification variable-by-sex, for example age*sex.

b. Information processing speed

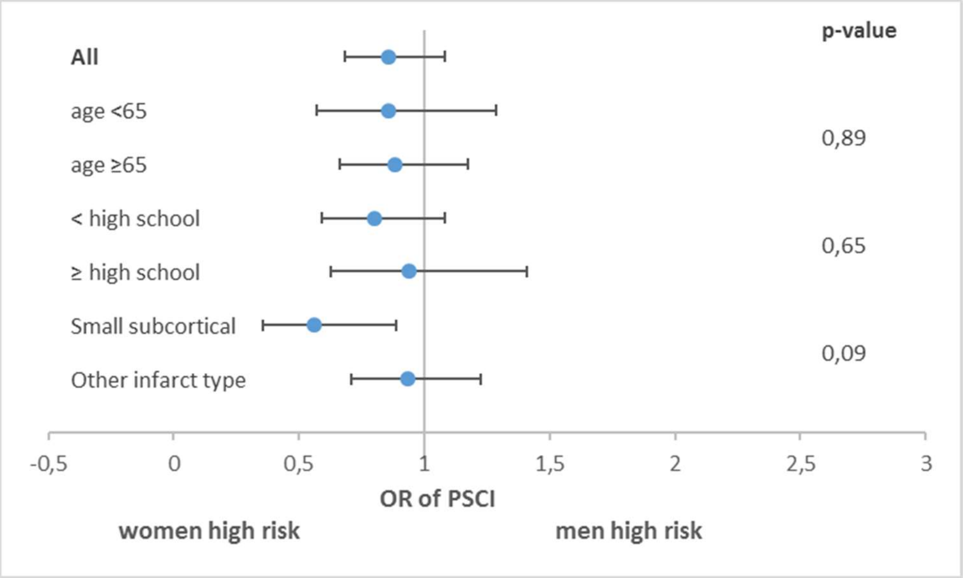

N=2091; missing 252 (11%)
Logistic regression analyses were performed to obtain OR for PSCI; females denoted the reference group.
The p-values depict the p-value of the interaction term stratification variable-by-sex, for example age*sex.

c. Language

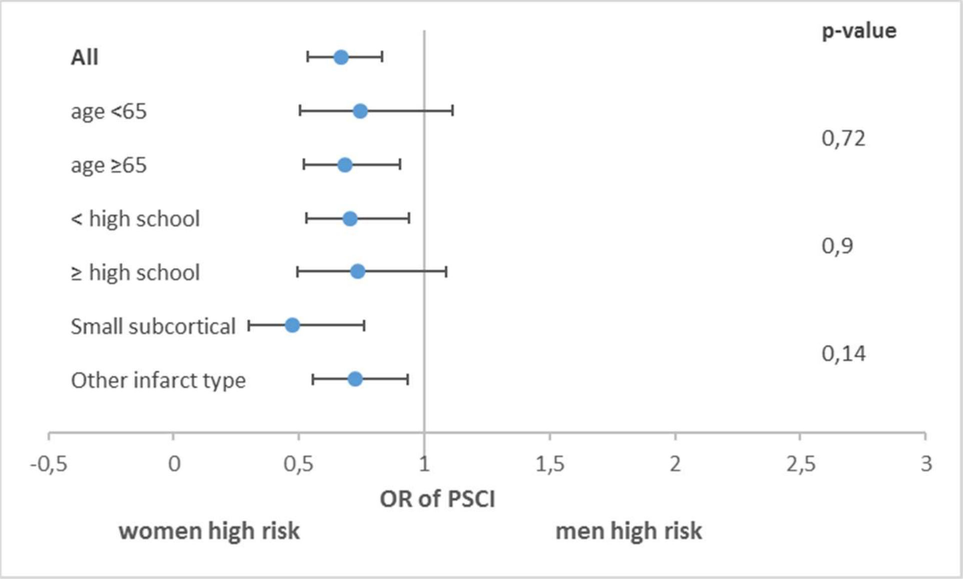

N=2304; missing 39 (2%)
Logistic regression analyses were performed to obtain OR for PSCI; females denoted the reference group.
The p-values depict the p-value of the interaction term stratification variable-by-sex, for example age*sex.

d. Verbal memory

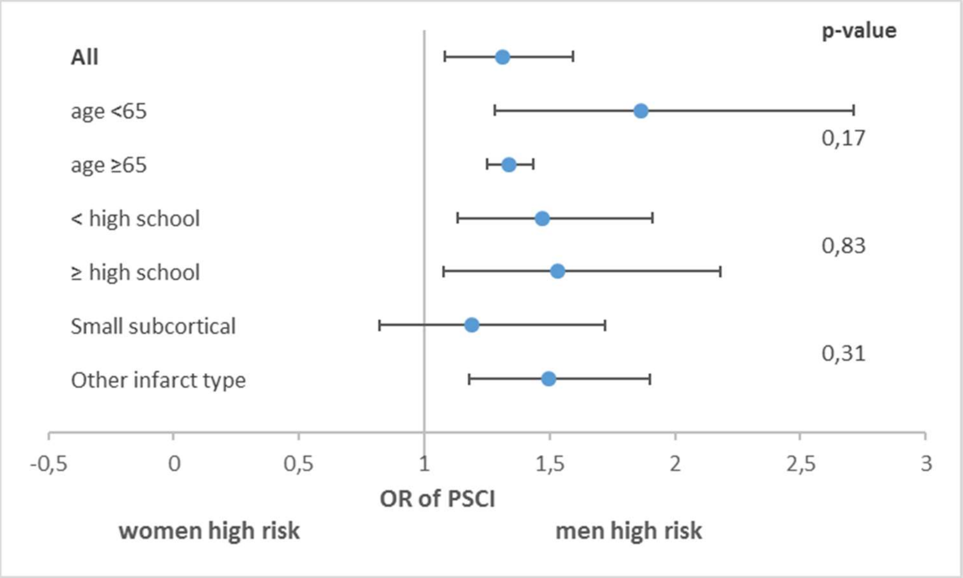

N=2286; missing 57 (2%)
Logistic regression analyses were performed to obtain OR for PSCI; females denoted the reference group.
The p-values depict the p-value of the interaction term stratification variable-by-sex, for example age*sex.

e. Visuospatial perception/construction

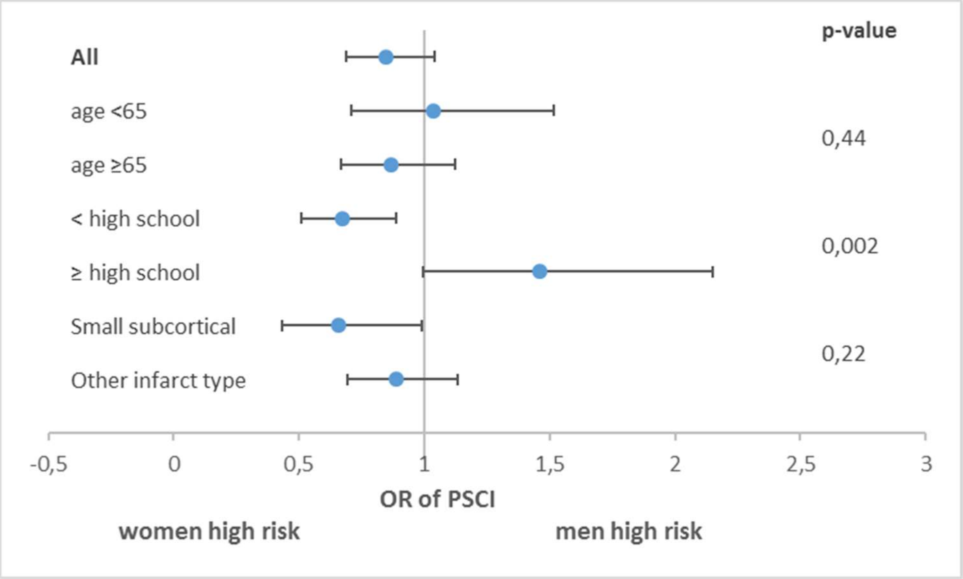

N=1875; missing 468 (20%)
Logistic regression analyses were performed to obtain OR for PSCI; females denoted the reference group.
The p-values depict the p-value of the interaction term stratification variable-by-sex, for example age*sex.

f. visuospatial memory

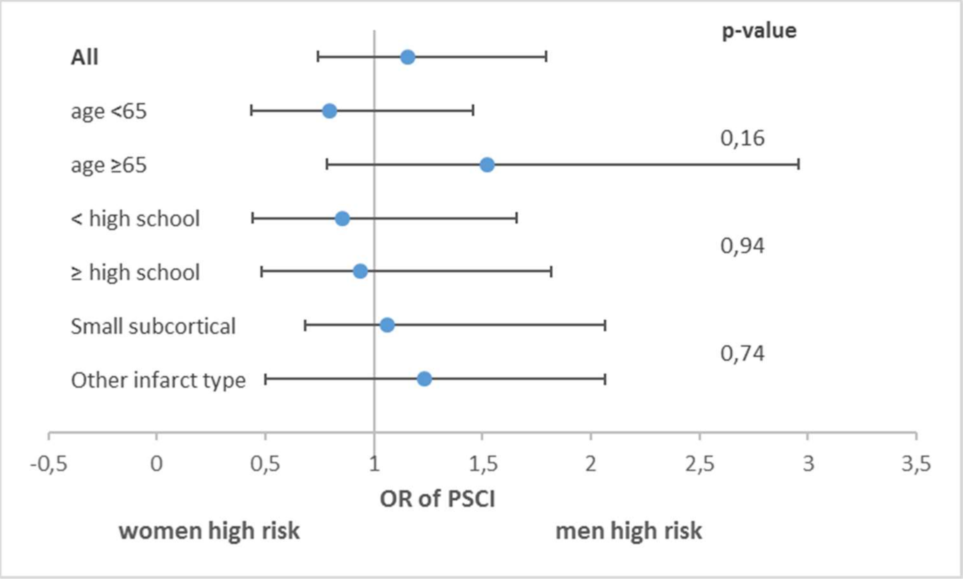

N=806; missing 1537 (66%)
Logistic regression analyses were performed to obtain OR for PSCI; females denoted the reference group.
The p-values depict the p-value of the interaction term stratification variable-by-sex, for example age*sex.

Figure S3. ROC curve of sensitivity and specificity of MoCA and MMSE by Sex.

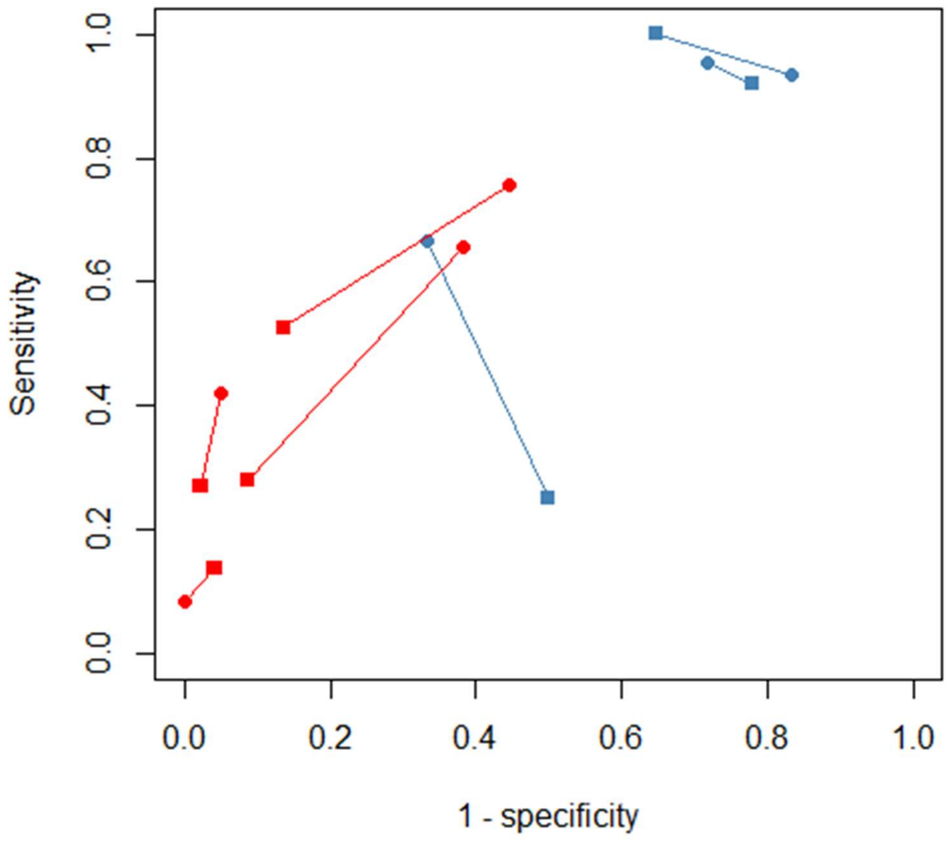

Blue = MoCA (cut-off <26), red = MMSE (cuf-off <25). Circles = women, squares = men.

Table S6 a. Bivariate analysis of MMSE data with sex as a covariate.

	Estimate (logit scale)	Lower CL (logit scale)	Upper CL (logit scale)	P value	Sens/spec	Lower CL	Upper CL
Women sens	0.125	-1.682	1.931		0.531	0.157	0.873
Women spec	1.405	-1.13	3.944		0.803	0.244	0.981
Difference sens	-1.106	-1.723	-0.489	0.02			
Difference spec	1.672	0.801	2.544	0.01			
Men sens	-0.982	-2.774	0.811		0.273	0.059	0.692
Men spec	3.078	0.494	5.663		0.956	0.621	0.997

Table S6 b. Sensitivity univariate analysis of MoCA data with sex as a covariate, including CODECS study

	Estimate (logit scale)	Lower CL (logit scale)	Upper CL (logit scale)	P value	Sens	Lower CL	Upper CL
Women sens	2.300	-1.991	6.591		0.909	0.120	0.999
Men Sens	1.853	-2.181	5.886		0.864	0.101	0.997
Difference	-0.447	-3.786	2.891	0.62			

Table S6 c. Specificity univariate analyses of MoCA data with sex as a covariate, including CODECS study.

	Estimate (logit scale)	Lower CL (logit scale)	Upper CL (logit scale)	P value	Spec	Lower CL	Upper CL
Women spec	-0.882	-2.359	0.595		0.293	0.086	0.644
Men spec	-0.965	-1.859	-0.071		0.276	0.135	0.482
Difference	-0.083	-1.809	1.644	0.86			

As the CODECS cohort is an outlier among the MoCA cohorts, probably because the cohort included isolated cerebellar stroke only, the analyses below are repeated without this cohort, showing similar results.

Table S6 d. Sensitivity univariate analysis of MoCA data with sex as a covariate, excluding CODECS study

	Estimate (logit scale)	Lower CL (logit scale)	Upper CL (logit scale)	P value	Sens	Lower CL	Upper CL
Women sens	2.833	-6.412	12.078		0.944	0.002	0.999
Men Sens	2.813	-4.739	10.366		0.943	0.009	0.999
Difference	-0.020	-11.958	11.918	0.99			

Table S6 e. Specificity univariate analyses of MoCA data with sex as a covariate, excluding CODECS study.

	Estimate (logit scale)	Lower CL (logit scale)	Upper CL (logit scale)	P value	Spec	Lower CL	Upper CL
Women spec	-1.030	-5.711	3.651		0.263	0.003	0.975
Men spec	-1.027	-3.777	1.723		0.264	0.022	0.848

Table S7. Sensitivity and specificity of MoCA and MMSE for PSCI by sex.

	PSCI		no PSCI	
	**Women**	**Men**	**Women**	**Men**
	(n=464)	(n=724)	(n=449)	(n=706)
**MoCA**	42	57	41	116
**MoCa score, mean (SD)**	18.5 ±4.7	20.1 ±5	24.1 ±3.4	23.3 ±3.4
**MoCA <26, n (%)**	38 (91%)	51 (90%)	29 (71%)	84 (72%)
**MMSE**	376	611	346	480
**MMSE score, mean (SD)**	20.7 ±6.7	24.0 ±5.6	25.2 ±4.3	27.6 ±2.4
**MMSE <25, n (%)**	238 (63%)	240 (39%)	122 (35%)	41 (9%)

MoCa = Montreal Cognitive Assessment, MMSE = Mini-Mental State Examination, SD = standard deviation.

Table S8. MoCA and MMSE score from cohorts without NTB.

	All	Women	Men	p-value
**STRIDE cohort [6,7]**	410	163	247	
Age, mean ±SD	68.6 ±10.4	71.3 ±10.3	66.8 ±10.1	<.000
Education low, n (%)	29 (7%)	6	23	.03
MMSE score	22.5 ±6.3	22.5 ±6.5	22.5 ±6.2	.44
MMSE <25	207 (51%)	78 (48%)	129 (52%)	.39
**CROMIS [5], Mild Stroke cohort [9] and STRIDE[6,7]**	607	244	363	
Age, mean ±SD	68.9 ±10.7	71.3 ±11.1	67.3 ±10.0	<.000
Education low, n (%)	201 (33%)	73 (30%)	128 (35%)	.17
MoCa score	21.3 ±5.8	19.2 ±6.3	22.7 ±5.0	<.000
MoCA <26	442 (73%)	204 (84%)	238 (66%)	<.000

Data are presented as mean ± SD or n (%).

Comparison between the sex strata by student T (mean), chi-square for n(%) are reported as p-value (unadjusted).
